# Multi-Scale Frequency Bands Ensemble Learning for EEG-Based Emotion Recognition

**DOI:** 10.3390/s21041262

**Published:** 2021-02-10

**Authors:** Fangyao Shen, Yong Peng, Wanzeng Kong, Guojun Dai

**Affiliations:** 1 School of Computer Science and Technology, Hangzhou Dianzi University, Hangzhou 310018, China; shenfangyao@hdu.edu.cn (F.S.); yongpeng@hdu.edu.cn (Y.P.); kongwanzeng@hdu.edu.cn (W.K.); 2 MoE Key Laboratory of Advanced Perception and Intelligent Control of High-End Equipment, Anhui Polytechnic University, Wuhu 241000, China; 3 Key Laboratory of Brain Machine Collaborative Intelligence of Zhejiang Province, Hangzhou 310018, China

**Keywords:** electroencephalography, emotion recognition, multi-scale, ensemble learning, frequency bands

## Abstract

Emotion recognition has a wide range of potential applications in the real world. Among the emotion recognition data sources, electroencephalography (EEG) signals can record the neural activities across the human brain, providing us a reliable way to recognize the emotional states. Most of existing EEG-based emotion recognition studies directly concatenated features extracted from all EEG frequency bands for emotion classification. This way assumes that all frequency bands share the same importance by default; however, it cannot always obtain the optimal performance. In this paper, we present a novel multi-scale frequency bands ensemble learning (MSFBEL) method to perform emotion recognition from EEG signals. Concretely, we first re-organize all frequency bands into several local scales and one global scale. Then we train a base classifier on each scale. Finally we fuse the results of all scales by designing an adaptive weight learning method which automatically assigns larger weights to more important scales to further improve the performance. The proposed method is validated on two public data sets. For the “SEED IV” data set, MSFBEL achieves average accuracies of 82.75%, 87.87%, and 78.27% on the three sessions under the within-session experimental paradigm. For the “DEAP” data set, it obtains average accuracy of 74.22% for four-category classification under 5-fold cross validation. The experimental results demonstrate that the scale of frequency bands influences the emotion recognition rate, while the global scale that directly concatenating all frequency bands cannot always guarantee to obtain the best emotion recognition performance. Different scales provide complementary information to each other, and the proposed adaptive weight learning method can effectively fuse them to further enhance the performance.

## 1. Introduction

**Figure 1:**
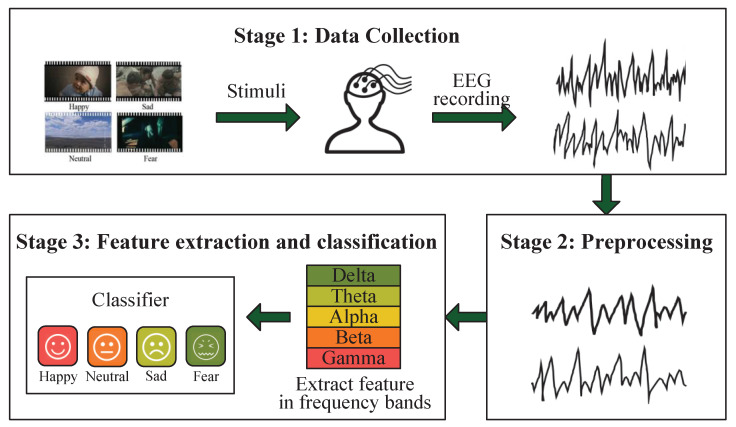
The flow chart of EEG-based emotion recognition system.

Developing automatic and accurate emotion recognition technologies has gained more and more attention due to its wide range of potential applications. In engineering, it facilitates the human–machine interaction more friendly, where machines might understand emotions and interact with us according to our emotions [1,2]. In the medical research, it is beneficial for diagnosing and treating various mental diseases, such as depression and autism spectrum disorders [3,4]. In the education field, it helps to track and improve the learning efficiency of students [5,6]. EEG signals record the neural activities of human cerebral cortex and reflect emotion states, providing an objective and reliable way to perform emotion recognition [7,8,9]. Besides, the advantages of EEG such as noninvasive, fast, and inexpensive in data acquisition make it become a preferred media in emotion recognition [10]. A popular video evoked EEG-based emotion recognition system is shown in Figure 1, which generally consists of the following stages. First, emotional video clips should be collected and subjects should be recruited before the experiments, and then EEG signals could be recorded from subjects who generate corresponding emotion states during watching emotional clips. Second, the raw EEG signals will be preprocessed including removing noise and filtering. Third, related features will be extracted and fed into a classifier to perform emotion classification. In this paper, we mainly focus on the last stage.

In the past decade, many feature extraction methods and classifiers were proposed for EEG-based emotion recognition [10]. Basically, EEG features can be divided into two types, the time-domain features and the frequency-domain features. The time-domain features aim to extract the temporal information from EEG, e.g., the fractal dimension feature [11], the Hjorth feature [12] and the higher order crossing feature [13]. For the frequency-domain features, researchers usually first filter EEG signals into several frequency bands, and then extract EEG features from each frequency band. The frequency interval of interest is 1–50 Hz which is usually partitioned into five frequency bands, Delta (1–4 Hz), Theta (4–8 Hz), Alpha (8–14 Hz), Beta (14–31 Hz), and Gamma (31–50 Hz). The frequency-domain features mainly include the differential entropy (DE) feature [14], the power spectral density (PSD) feature [15], the differential asymmetry (DASM) feature [11], the rational asymmetry (RASM) feature [16] and so on. Lu et al. made a detailed comparison among these features, and found that DE was the most stable and accurate feature for emotion recognition than the others [14,17]. Therefore, the DE feature is adopted in this paper. On the classifiers, many machine learning methods were proposed for EEG-based emotion recognition [18,19]. Peng et al. designed a discriminative manifold extreme learning machine (DMELM) method for emotion recognition, and found that Beta and Gamma frequency bands were more relevant to emotional states transition than the others [20]. Li et al. proposed a hierarchical convolutional neural network (HCNN) to classify emotion states, and their experimental results also indicated that the high frequency bands (Beta and Gamma) performed better than the low frequency bands [21]. Moreover, Zheng et al. introduced deep belief networks (DBNs) to construct EEG-based emotion recognition models, and their results demonstrated that combining all frequency bands together performed better than individual bands [22]. Yang et al. drew similar conclusion by designing a Continuous Convolutional Neural Network [23]. The potential reason was that the multiple bands could provide complementary information to each other.

**Figure 2:**
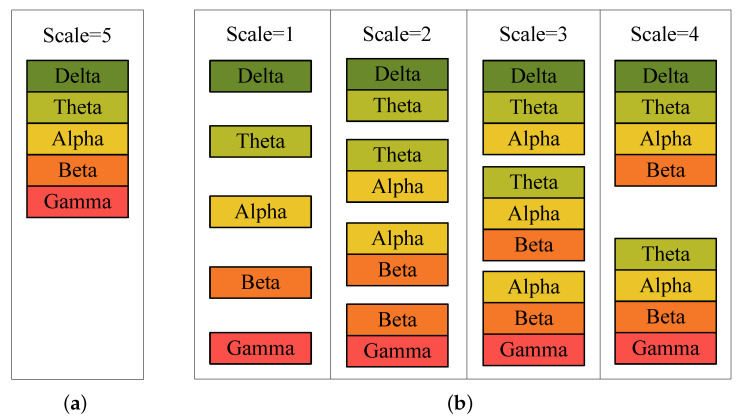
Different scales of frequency bands: (**a**) global scale, (**b**) local scales.

Although the methods mentioned above have achieved improvement in EEG-based emotion recognition, there still exists a problem. The way of frequency bands combination of them is directly concatenating all frequency bands together, which termed as the global scale in this paper and depicted in Figure 2a. However, such way cannot always achieve the best results since it essentially assumes that all frequency bands share the same importance. In this paper, we make extension on the way of combining frequency bands, which is termed as local scales and shown in Figure 2b. Here, by taking the face recognition task as an example, we illustrate the rationality of such multi-scale setting. Human faces manifest distinct characteristics and structures when observe in different scales, and different scales provide complementary information to each other [24]. Similarly, we assume that different scales of frequency bands hold different characteristics of emotion, as well as complement to each other. In each scale, we combine adjacent frequency bands into patches. For example, when the scale is 2, patches are formed by combining 2 adjacent frequency bands, and there are 4 patches in total. It should be noted that we only combine adjacent frequency bands into a patch because the frequency bands changing from Delta to Gamma reflects the conscious mind going from weak to active, which is a continuous process [10]. Therefore, it is reasonable to combine adjacent frequency bands.

For each scale, we train a base classifier to obtain the single-scale classification result. After that, the critical step is how to fuse the results of all scales to enhance the overall performance. It essentially belongs to an ensemble learning task [25,26,27], which combines the results of a set of base classifiers to perform better. In this paper, we design an adaptive weight learning method to combine all scales, which considers the classifier on each scale as a base classifier and learns the weight of each scale to fuse multi-scale results.

From the above, we propose a novel multi-scale frequency bands ensemble learning (MSFBEL) for EEG-based emotion recognition. Generally, the main contributions of this work are summarized as follows.

- We extended the way of combining different frequency bands into four local scales and one global scale, and then performed emotion recognition on every scale with a single-scale classifier.
- We proposed an effective adaptive weight learning method to ensemble multi-scale results, which can adaptively learn the respective weights of different scales according to the maximal margin criterion, whose objective can be formulated as a quadratic programming problem with the simplex constraint.
- We conducted extensive experiments on benchmark emotional EEG data sets, and the results demonstrated that the global scale that directly concatenating all frequency bands cannot always guarantee to obtain the best emotion recognition performance. Different scales provide complementary information to each other, and the proposed method can effectively combine these information to further improve the performance.

The rest of this paper is organized as follows. Section 2 presents the proposed method in detail. Section 3 displays the emotional EEG data sets, experiments, and results of the proposed method. Section 4 concludes the whole paper and presents the future work.

In this paper, vectors are written as boldface lowercase letters, and matrices are written as boldface uppercase letters. The trace of matrix A is represented by Tr(A). For a vector a, the $\ell_{2}$-norm of it is denoted by ${∥a∥}_{2}={\left(a^{T}a\right)}^{\frac{1}{2}}$, where $a^{T}$ is the transpose of a. a≥0 represents that every element of vector a is larger than or equal to zero. 1 and I represent a column vector that all elements are “1” and an identity matrix, respectively. I denotes an indicator function which takes the value of 1 when the condition is true, and 0 otherwise.

## 2. Method

**Figure 3:**
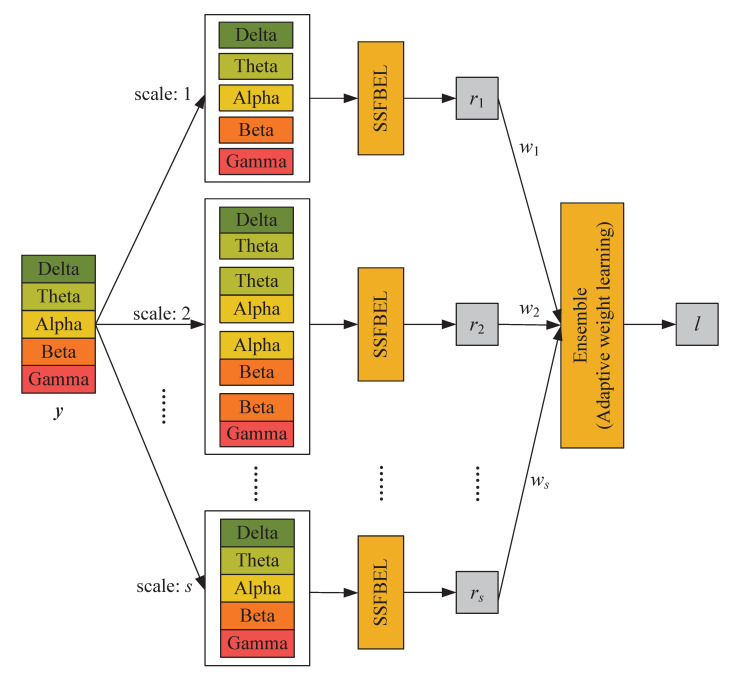
The framework of MSFBEL.

In this section, we present the proposed method MSFBEL (Figure 3) in detail, which mainly contains two stages. First, we re-arrange every EEG sample into different scales as shown in Figure 2, and then perform emotion classification on each scale by a single-scale classifier, called single-scale frequency band ensemble learning (SSFBEL). Second, the results of all scales are fused by the adaptive weight learning method to further improve the performance.

### 2.1. Single-Scale Frequency Band Ensemble Learning

In this paper, we use DE feature to model emotion information from EEG signals. Without loss of generality, supposing that DE features are extracted from *s* frequency bands, we divide it into *s* scales. In each scale *j* (j=1,2,⋯,s), we combine *j* adjacent frequency bands into patches and then obtain $p_{j}=s-j+1$ patches of this scale. Figure 2 displays an example when s=5, which represents that DE features are extracted from 5 frequency bands (Delta, Theta, Alpha, Beta, and Gamma).

**Figure 4:**
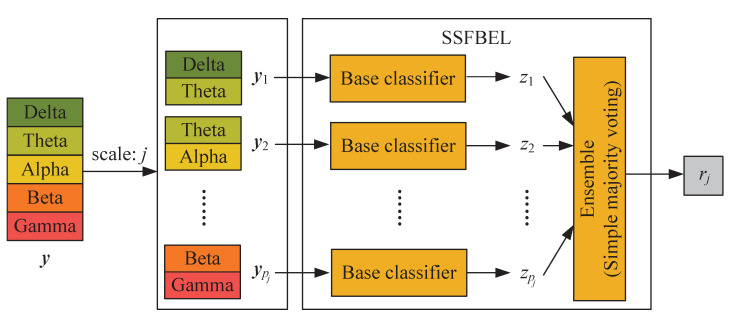
The architecture of SSFBEL.

SSFBEL is proposed to perform emotion classification on each scale of frequency bands, whose architecture is displayed in Figure 4, which takes an example with s=5 and j=2. Below we give some explanations of it.

- First, given an unlabeled DE feature-based sample $y\in R^{d}$, we divide it into a set of patches $y_{i}\in R^{d_{j}}\left(i=1,2,\cdots ,p_{j}\right)$. Here *d* is the feature dimension of EEG samples. For example, if we use the DE-based EEG feature representation, *d* is equal to the product of the numbers of channels and frequency bands. Similarly, $d_{j}$ denotes the feature dimension of DE patches under scale *j*, that is, $d_{j}$ equals the product of the numbers of channels and frequency bands in a patch.
- Second, these patches are, respectively fed into base classifiers and then the corresponding predicted labels $\left\{z_{1},z_{2},\cdots ,z_{p_{j}}\right\}$ can be obtained.
- Finally, the predicted labels of all patches are combined by simple majority voting [28] to generate the final label $r_{j}$ for the sample y under scale *j*.

In SSFBEL, the collaborative representation based classification (CRC) [29] is used as the base classifier. CRC usually represents a test sample with an over-complete dictionary formed by training samples, whose representation coefficient vector is regularized with an $\ell_{2}$-norm to improve its computational efficiency. Once the representation coefficient vector is obtained, the test sample can be categorized into the class which yields the minimum reconstruction error. In current study, for each patch, we construct the corresponding dictionary according to the principle that the combination and order of frequency bands of them are consistent.

Suppose that we have a patch $y_{i}$ and the corresponding dictionary formed by training samples $X_{i}=\left[X_{i1},X_{i2},\cdots ,X_{ic}\right]\in R^{d_{j}\times n}$, where *c*, $d_{j}$, and *n* are the number of classes, the feature dimension of DE patches under scale *j*, and the number of training samples, respectively. $X_{ik}\in R^{d_{j}\times n_{k}}$ (k=1,2,⋯,c) is the collection of samples from the *k*-th class in which each column is a sample, where $n_{k}$ denotes the number of samples in the *k*-th class. For the patch $y_{i}$, its representation coefficient $\alpha_{i}\in R^{n}$ can be obtained by solving the following objective

(1) $$\min_{\alpha_{i}}∥X_{i}\alpha_{i}-y_{i}{∥}_{2}^{2}+\lambda {∥\alpha_{i}∥}_{2}^{2},$$

where λ is a regularization parameter. Obviously, the optimal representation coefficient to (1) is

(2) $$\alpha_{i}^{*}={\left(X_{i}^{T}X_{i}+\lambda I\right)}^{-1}X_{i}^{T}y_{i}.$$

Let $\alpha_{ik}^{*}\in R^{n_{k}}$ represent the vector whose only nonzero entries are the entries of $\alpha_{i}^{*}$ associated with class *k*. The sample $y_{i}$ can be reconstructed by the training samples of class *k* as $y_{ik}=X_{ik}\alpha_{ik}^{*}$. The label of $y_{i}$ is determined as the class which yields the minimum reconstruction error

(3) $$z_{i}=\underset{k}{\arg\min}\;{∥X_{ik}\alpha_{ik}^{*}-y_{i}∥}_{2}.$$

In our experiments, to further improve the classification accuracy, we divided the above reconstruction error by $∥\alpha_{ik}^{*}{∥}_{2}$, because it can bring some discrimination information for classification [29]. Finally, the single-scale result $r_{j}$ is obtained, which combines all patches’ results $z_{i}$ ($i=1,2,\cdots ,p_{j}$) by using majority voting $r_{j}={\arg\max}_{k\in [c]}\left\{\sum_{i=1}^{p_{j}}I\left(z_{i}=k\right)\right\}$.

### 2.2. Adaptive Weight Learning

Assuming that different scales of frequency bands might have complementary information to each other, we combine the classification results of all scales obtained by SSFBEL to enhance the emotion recognition performance. The whole architecture of the proposed MSFBEL is shown in Figure 3.

Obviously, the current task is how to determine the optimal weights for different scales in the ensemble stage. In this work, we propose an adaptive weight learning method by maximizing the ensemble margin. Suppose the ensemble weight vector is $w=\left[w_{1};w_{2};\cdots ;w_{s}\right]\in R^{s}$, where $\sum_{j=1}^{s}w_{j}=1$, and we learn it from data. Specifically, we select *m* samples from the total *n* training samples, whose data and labels can be, respectively represented as $X_{sub}=\left[x_{1},x_{2},\cdots ,x_{m}\right]\in R^{d\times m}$ and $O_{sub}=\left[o_{1},o_{2},\cdots ,o_{m}\right]\in R^{c\times m}$, where $x_{p}\in R^{d}$ (p=1,2,⋯,m) represents the *p*-th DE-based EEG sample, $o_{p}$ is the corresponding ground-truth label. We divide a sample $x_{p}$ into *s* scales and feed them into SSFBEL to obtain the classification result of each scale $r_{pj}$ (j=1,2,⋯,s). We define the decision matrix $D=\left[d_{pj}\right]\in R^{m\times s}$ as

(4) $$d_{pj}=\left\{\begin{array}{cc} +1, & \;\mathrm{if}\;o_{p}=r_{pj}\\ -1, & \;\mathrm{if}\;o_{p}\neq r_{pj}. \end{array}\right.$$

Then, the ensemble margin of sample $x_{p}$ is defined as

(5) $$\varepsilon \left(x_{p}\right)=\sum_{j=1}^{s}w_{j}d_{pj}.$$

To get the optimal weight vector w, we should make the ensemble margin in (5) as large as possible. Based on the studies of [30,31], margin maximization can be transformed into a loss minimization problem. To be specific, the ensemble loss function can be formulated as

(6) $$\begin{array}{cc} loss & =\sum_{p=1}^{m}\left[1-\varepsilon \left(x_{p}\right)\right]\\ & =\sum_{p=1}^{m}\left[1-\sum_{j=1}^{s}w_{j}d_{pj}\right]\\ & =∥1_{m}{-\mathrm{Dw}∥}_{2}^{2}, \end{array}$$

where $1_{m}=\left[1;1;\cdots ;1\right]\in R^{m}$ is a column vector. Therefore, objective (6) is equivalent to optimize

(7) $$\min_{w\geq 0,w^{T}1_{s}=1}{∥1_{m}-\mathrm{Dw}∥}_{2}^{2},$$

where $1_{s}=\left[1;1;\cdots ;1\right]\in R^{s}$ is a column vector. By denoting $M=D^{T}D$ and $b=2D^{T}1_{m}$, we can rewrite the objective (7) as

(8) $$\begin{array}{cc} \; & \min_{w\geq 0,w^{T}1_{s}=1}\mathrm{Tr}\left({\left(1_{m}-\mathrm{Dw}\right)}^{T}\left(1_{m}-\mathrm{Dw}\right)\right)\\ \Leftrightarrow & \min_{w\geq 0,w^{T}1_{s}=1}\mathrm{Tr}\left(1_{m}^{T}1_{m}-2w^{T}D^{T}1_{m}+w^{T}D^{T}\mathrm{Dw}\right)\\ \Leftrightarrow & \min_{w\geq 0,w^{T}1_{s}=1}\mathrm{Tr}\left(w^{T}D^{T}\mathrm{Dw}-2w^{T}D^{T}1_{m}\right)\\ \Leftrightarrow & \min_{w\geq 0,w^{T}1_{s}=1}\mathrm{Tr}\left(w^{T}Mw-w^{T}b\right). \end{array}$$

Since w is a column vector, objective (8) is equivalent to optimize

(9) $$\min_{w\geq 0,w^{T}1_{s}=1}w^{T}Mw-w^{T}b.$$

To make the above objective separable, we introduce an auxiliary variable v with respect to w, then we get

(10) $$\min_{w\geq 0,w^{T}1_{s}=1}v^{T}Mw-v^{T}b.$$

According to the augmented Lagrangian multiplier (ALM) method [32,33], objective (10) can be rewritten as

(11) $$\min_{w\geq 0,w^{T}1_{s}=1,v}v^{T}Mw-v^{T}b+\frac{\mu }{2}{∥w-v+\frac{\beta }{\mu }∥}_{2}^{2},$$

where μ is a quadratic penalty parameter and $\beta \in R^{s}$ is the Lagrangian multiplier.

Accordingly, an alternative optimization method is applied to solving problem (11). The details are given in Appendix A. Finally, we get the optimum solution $w^{*}$ to problem (11), based on which we can make prediction on the test sample y as $l={\arg\max}_{k\in [c]}\{\sum_{j=1}^{s}w_{j}^{*}I$$\left(r_{j}=k)\right\}$. The procedure of MSFBEL framework is outlined in Algorithm 1.
**Algorithm 1** The procedure for MSFBEL framework.**Input:** Number of scales *s*, number of classes *c*, training data $X\in R^{d\times n}$, training data label   $O\in R^{c\times n}$, a subset of training data $X_{sub}\in R^{d\times m}$, the labels of subset $O_{sub}\in R^{c\times m}$,   testing data $y\in R^{d}$;
**Output:** The label of testing data: *l*.
 1: **for**
j=1:s
**do**
 2:  Compute the label of testing data under scale *j* via Algorithm 2;
 3: **end for**
 4: Compute decision matrix D by Equation (4) with $X_{sub}$ and $O_{sub}$;
 5: Compute $M=D^{T}D$ and $b=2D^{T}1_{m}$; 6: Compute the adaptive weight $w^{*}$ via Algorithm 3; 7: Compute $l={\arg\max}_{k\in [c]}\left\{\sum_{j=1}^{s}w_{j}^{*}I\left(r_{j}=k\right)\right\}$.


## 3. Experiments and Results

In this section, we first describe two emotional EEG data sets used in the experiments, including their data collection and feature extraction. Then, the experimental settings are given based on which we perform EEG-based emotion recognition to evaluate the effectiveness of MSFBEL.
**Algorithm 2** The procedure for SSFBEL framework.**Input:***s*, *c*, λ, $X_{i}=\left[X_{i1},X_{i2},\cdots ,X_{ic}\right]\in R^{d_{j}\times n}$, $y_{i}\in R^{d_{j}}$, where $i=1,2,\cdots ,p_{j}$ and    $p_{j}=s-j+1$; **Output:** The label of testing data under scale *j*: $r_{j}$.  1: Compute $\alpha_{i}^{*}$ by Equation (2);   2: Compute $z_{i}$ by Equation (3);   3: Compute $r_{j}={\arg\max}_{k\in [c]}\left\{\sum_{i=1}^{p_{j}}I\left(z_{i}=k\right)\right\}$.


**Algorithm 3** The algorithm to solve problem (11).**Input:**M and b;
**Output:** The weight vector $w\in R^{s}$.  1: Initialize v, β, μ, and ρ;  2: **while** not converged **do**
  3:  Update v by Equation (A1);  4:  Update w by solving problem (A3) via Algorithm 4;  5:  Update β=β+μ(w−v);  6:  Update μ=ρμ;  7: **end while**


**Algorithm 4** The algorithm to solve problem (A3).**Input:**M, v, β, μ and *s*; **Output:** The weight vector $w\in R^{s}$.  1: Compute $g=v-\frac{\beta +M^{T}v}{\mu }\in R^{s}$;  2: Compute $h=g-\frac{1_{s}^{T}g}{s}1_{s}+\frac{1}{s}1_{s}$;  3: Use Newton’s method to obtain the root ${\bar{\eta }}^{*}$ of Equation (A12);  4: The optimal solution $w_{j}^{*}={\left(h_{j}-{\bar{\eta }}^{*}\right)}_{+}$, where j=1,2,⋯,s.


### 3.1. Data Set

#### 3.1.1. SEED IV

The “SEED IV” is a publicly available emotional EEG data set [34]. The EEG signals were collected from 15 healthy subjects when they watched emotion-eliciting videos. In the EEG data collection experiment, 24 two-minute video clips were played to each subject. There are four types of emotional video clips, sadness, fear, happiness, and neutral, and each emotion has 6 video clips. While watching video clips, EEG signals of subjects were recorded at a 1000 Hz sampling rate by the 62-channel ESI NeuroScan system (https://compumedicsneuroscan.com/ (accessed on 6 August 2020)). Every subject was asked to complete the experiment three times on different days, and therefore we obtained three sessions of EEG signals for each subject.

In our experiments, we used the “EEG_feature_smooth” EEG data recordings downloaded from the “SEED IV” web site (http://bcmi.sjtu.edu.cn/home/seed/seed-iv.html (accessed on 20 December 2019)). Preprocessing and feature extraction of EEG data had already been conducted, including downsampling all data to 200 Hz, filtering out noise and artifacts by linear dynamic system (LDS) [35], and extracting DE features from 5 frequency bands: Delta, Theta, Alpha, Beta, and Gamma, with a four-second time window without overlapping. The dimensions of DE feature were $62\times W_{1}\times 5$ (format: # channel × # samples × # frequency bands), where $W_{1}$ was the number of samples of one subject in each trial. We reshaped the 62 points of each of the 5 frequency bands, and then obtained DE features with the shape of $310\times W_{1}$. Since the time durations of different video clips in each session were slightly different, the total sample numbers for each subject in each session were approximately 830.

#### 3.1.2. DEAP

Another emotional EEG data set used to validate our proposed method is “DEAP” [36]. It is a music video evoked EEG data set. There were 32 subjects invited to watch 40 one-minute music video clips. At the end of each video, subjects were asked to make a self-assessment of their level in terms of arousal, valence, liking, and dominance. During the experiment, the EEG signals were recorded by Biosemi ActiveTwo system (http://www.biosemi.com (accessed on 6 August 2020) with 32-channel electrode according to the international 10–20 system placement. The sampling rate is 512 Hz.

In our experiments, we utilized the “Data_preprocessed_matlab” EEG data recordings downloaded from the “DEAP” web site (http://www.eecs.qmul.ac.uk/mmv/datasets/deap/index.html (accessed on 20 December 2019)). EEG signals were down-sampled to 128 Hz. EOG artefacts were removed by using a blind source separation technique (http://www.cs.tut.fi/gomezher/projects/eeg/aar.htm (accessed on 6 August 2020)). A bandpass frequency filter from 4.0 to 45.0 Hz was applied. Then, we extracted DE features from 4 frequency bands: Theta, Alpha, Beta, and Gamma, with a one-second window size without overlapping. The dimensions of DE feature were $32\times W_{2}\times 4$, where $W_{2}$ was the number of samples in each trial. We concatenated the 32 values of the 4 frequency bands and then obtained DE features with the shape of $128\times W_{2}$. We got 63 samples for each trial in which the first 3 samples were baseline signals, and the last 60 sample were trial signals. According to the study of Yang et al. [37] that the baseline signals were useful for emotion recognition, we further processed the data as they did: calculating the deviation between every trial sample and the average of 3 baseline samples as the final input. Therefore, we obtained 60 samples in each trial, and there were totally 2400 samples for each subject.

### 3.2. Experimental Settings

We evaluated methods on every subject of the two data sets. For the “SEED IV” data set, we evaluated methods under the within-session experimental paradigm as [34]. Specifically, for every subject in each session, we utilized the last 8 trials as the test data, which not only contained all emotional states but also guaranteed that each emotional state has exact 2 trials, and the rest 16 trials as the training data. On the “DEAP” data set, we split every subject’s data as [36], which chose 5 as a threshold to divide all samples into four categories according to the different levels of valence and arousal: high valence and high arousal (HVHA), high valence and low arousal (HVLA), low valence and high arousal (LVHA), low valence and low arousal (LVLA). Then we performed 5-fold cross validation on each subject’s data. The recognition accuracy and standard deviation were used as evaluation metrics. The average accuracy and standard deviation of all subjects represent the final performance of a method.

We compare MSFBEL with support vector machine (SVM), *K* Nearest Neighbors (KNN) and SSFBEL. Linear kernel was used in SVM and the regularization parameter *C* was determined by grid search from $\left\{10^{-5},10^{-4},\cdots ,10^{5}\right\}$. For KNN, Euclidean distance measure was used and the number of neighboring samples *K* was searched from {10,20,30,⋯,150}. The regularization parameter λ in objective (2) of SSFBEL was fine-tuned with grid search from $\left\{10^{-5},10^{-4},\cdots ,10^{-1}\right\}$. The parameters μ in objective (11) of MSFBEL was fine-tuned with grid search from $\left\{10^{-5},10^{-4},\cdots ,10^{-1}\right\}$, β and ρ were initialized with $\left[1;1;\cdots ;1\right]\in R^{s}$ and 1.1, respectively. To learn the optimal weights corresponding to different scales, we need to get the decision matrix D defined in Equation (4). Therefore, we divided the training data into two parts; one was used for training and the other was used for validation. For the “SEED IV” data set, each part contained 2 trials of each emotional state. For the “DEAP” data set, each part contained half samples of each emotional category. Then we calculated the decision matrix D based on the ground truth labels and the estimated labels of samples in the validation set.

### 3.3. Experimental Results and Analysis

#### 3.3.1. The Effect of Different Scales

**Figure 5:**
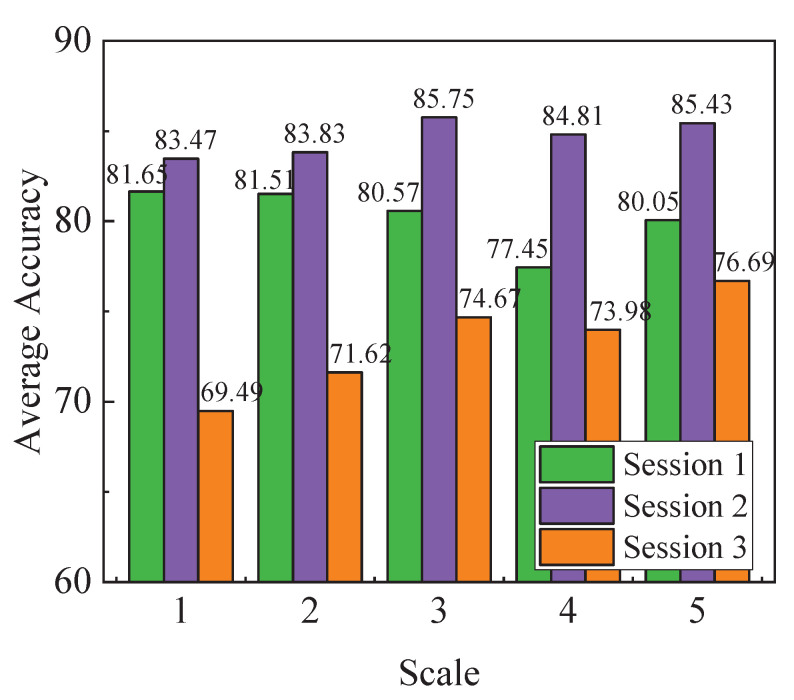
The average accuracies of different scales of frequency bands on the three sessions of “SEED IV” by using SSFBEL.

In this section, we compare the performance of global scale and local scales of frequency bands for emotion recognition by SSFBEL. First, we perform classification on different scales for every subject’ data from the “SEED IV” and “DEAP” data sets, and the results are shown in Table 1, Table 2, Table 3 and Table 4, respectively. The best results are highlighted in boldface. From these tables, we can observe that not every subject gets the best results on the global scale (scale = 5 for “SEED IV”, and scale = 4 for “DEAP”). For example, for subject #1 in Table 1, SSFBEL gets the best accuracy 84.85% when scale = 1, which exceeds the accuracy of scale = 5 by 1.17%. For subject #6, it obtains the optimal result 79.25% when scale = 2, which outperforms the result of scale = 5 by 12.58%. We can find similar results from the other three tables. Second, we calculate the average accuracy of all subjects in each scale of frequency bands. The average accuracies of each scale on the three sessions of “SEED IV” data set are shown in Figure 5. From this figure, we can find that the global scale cannot always achieve the optimal accuracy on every session. For example, in session 1, SSFBEL achieves the highest average accuracy 81.65% on scale = 1, which is higher than that of scale = 5 by 1.46%. These results are consistent with our proposed idea that the global scale of frequency bands which direct concatenating all frequency bands together cannot always achieve the best performance. That is, sometimes local scales can achieve higher emotion recognition accuracy than the global scale. Therefore, it is reasonable for us to re-organize all frequency bands into different scales, which can provide more potential for improving the performance of emotion recognition.

However, there still exists a problem that the optimal scale of frequency bands varies in terms of different subjects. For example, in Table 1, some subjects (#1, #3, #4, #7, #9, #10, #12, and #15) get the best result on scale = 1, and some subjects (#2, #6, #9, and #14) obtain the optimal result on scale = 2, and so on. Besides, some subjects (#2 and #9) achieve the best result on several scales at the same time. These problems can be found in the other three tables. The uncertainty of the optimal scale may be influenced by the characteristics of the EEG signals, which not only have low signal-to-noise ratio (SNR) but also exhibit significant differences across subjects [38]. Therefore, it is necessary to fuse all scales to reduce the impact of these factors. In this paper, MSFBEL is proposed to fuse different scales, in which the adaptive weight learning can fuse all scales’ results through automatically assigning larger weights to more important scales to further enhance the performance. The effectiveness of MSFBEL will be evaluated in Section 3.3.2.

#### 3.3.2. The Performance of MSFBEL

In this part, we compare MSFBEL with SVM, KNN and SSFBEL to show the effectiveness of it. In this experiment, SVM and KNN take global scale frequency bands as input. As for SSFBEL, we choose the best frequency band scale for every data set as input. Specifically, according to the average accuracies shown in Table 1, Table 2, Table 3 and Table 4, we choose the results of 1, 3, and 5 for session 1, 2, and 3 of the “SEED IV” data set, and we select the results of scale = 4 for the “DEAP” data set. MSFBEL method takes all frequency band scales as input.

**Figure 6:**
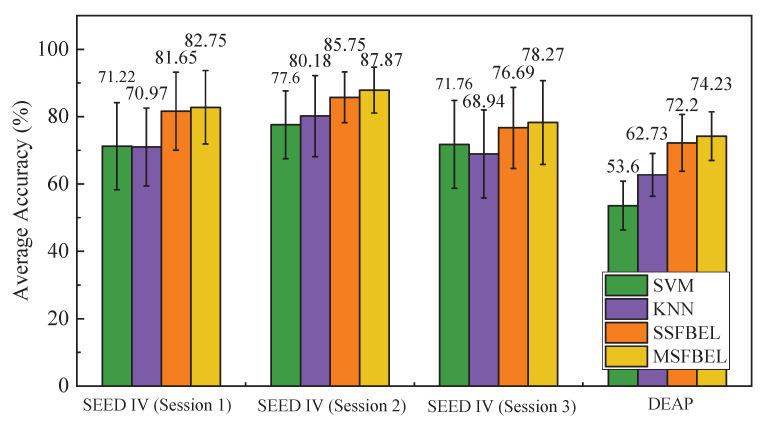
The average accuracies of different methods on the two data sets for EEG-based emotion recognition.

For the “SEED IV” data set, emotion recognition accuracies of the four methods in the three sessions are shown in Table 5. From the experimental results, we observe that MSFBEL achieves the best average recognition rates of 82.75%, 87.87%, and 78.27% in the three sessions, respectively. When compared with SVM, MSFBEL respectively achieves 11.53%, 10.27% and 6.51% improvements in the three sessions. As for KNN, MSFBEL respectively obtains 11.78%, 7.69% and 9.33% improvements than it in the three sessions. Moreover, MSFBEL exceeds SSFBEL by 1.10%, 2.12%, and 1.57% corresponding to the three sessions. For the “DEAP” data set, accuracies of the four methods are displayed in Table 6. From this table, we can find that MSFBEL achieves the average accuracy of 74.23%, which obtains 20.63% and 11.5% improvements in comparison with SVM and KNN. In addition, the performance of MSFBEL is better than SSFBEL by 2.03% in terms of average accuracy. Besides, Figure 6 presents the overall performance of the four methods. We observe that MSFBEL achieves better performance than the other three methods on both data sets. The underlying reason may be the combination of different scales of frequency bands by assigning larger weights to important scales by MSFBEL, while the other three methods only conduct classification on one scale. Therefore, we declare that there are complementary information among different scales, and the proposed adaptive weight learning method effectively fuses these information to enhance classification performance.

**Figure 7:**
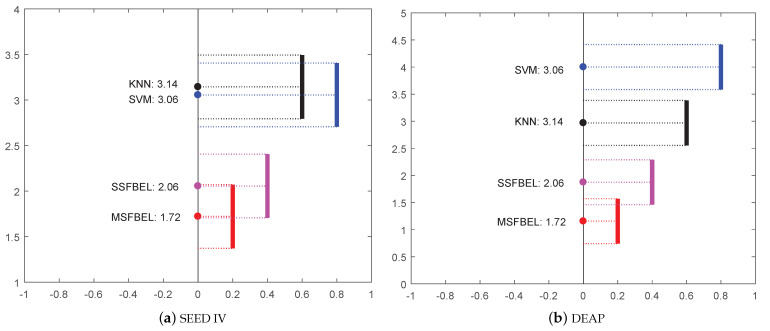
Friedman test of different methods on the two data sets.

Besides the comparison of average accuracy of these four methods, we perform the Friedman test [39] to illustrate the statistical significance among them. The Friedman test is a non-parametric statistical test, which is used to detect differences of multiple methods across multiple test results. The null-hypothesis is “all the methods have the same performance”. If the null-hypothesis is rejected, the Nemenyi test is utilized to further distinguish whether the performances of the two among all methods are significantly different. We analyze the difference in performance among the four methods, and the results are shown in Figure 7. In the figure, the solid circle represents the average rank of each method, and the vertical line represents the critical distance (CD) of Nemenyi test, which is defined as follows

(12) $$CD=q_{\alpha }\sqrt{\frac{k(k+1)}{6N}}.$$

where *k* denotes the number of methods, *N* denotes the number of result groups, and $q_{\alpha }$ is the critical value which is defaulted as 0.05 [40]. In this paper, we set k=4 because there are four methods in total. For “SEED IV” data set, N=45 because there are 15 subjects and each subject has 3 sessions. For “DEAP” data set, N=32 since there are 32 subjects. If two vertical lines do not have overlap, it means that the corresponding methods have statistically different performance. As shown in Figure 7, both on “SEED IV” and “DEAP” data set, SSFBEL and MSFBEL do not have overlap with SVM and KNN, which represents that our proposed methods are significantly different in performance with these two compared methods.

**Figure 8:**
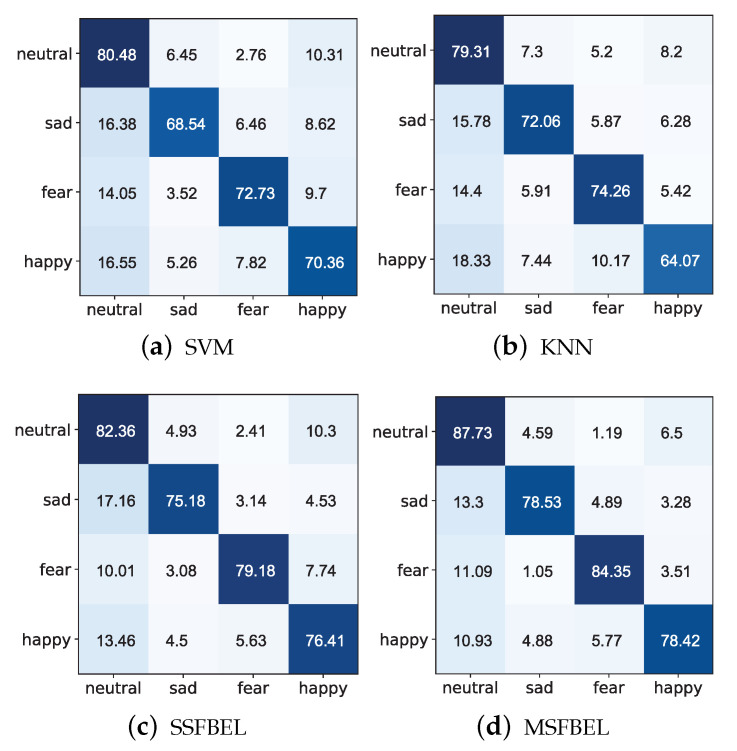
Confusion matrices of different methods on the “SEED IV” data set.

**Figure 9:**
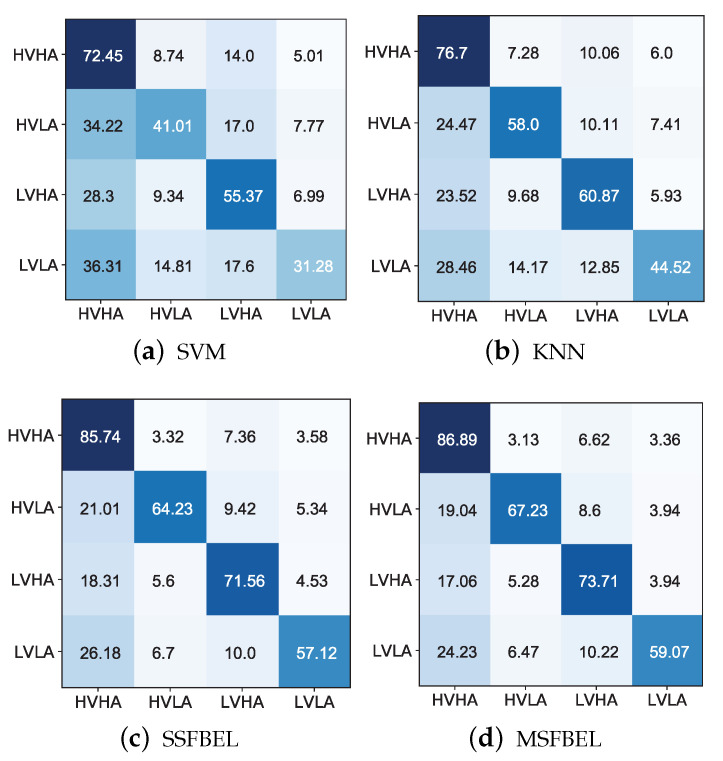
Confusion matrices of different methods on the “DEAP” data set.

Figure 8 and Figure 9 present the confusion matrices for the emotion recognition results of SVM, KNN, SSFBEL, and MSFBEL on the two data sets, respectively. From them, we first can obtain the average recognition accuracies of every emotional state. For example, in Figure 8, the average accuracy of the “neutral” emotional state classified by SVM, KNN, SSFBEL, and MSFBEL are 80.48%, 79.31%, 82.36%, and 87.73%, respectively. In Figure 9, the average accuracies of the “HVHA” emotional state classified by these four methods are 72.45%, 76.7%, 85.74%, and 86.89%, respectively. Second, we can get the misclassification rate of each emotion state. For example, from the confusion matrix of MSFBEL on the “SEED IV” data set (Figure 8d), 87.73% of the EEG samples are correctly recognized as “neutral” state while 4.59%, 1.19%, 6.5% of them are incorrectly classified as “sad”, “fear”, and “happy” states, respectively. From the confusion matrix of MSFBEL on the “DEAP” data set (Figure 9d), 86.89% of the EEG samples are correctly classified as “HVHA” state while 3.13%, 6.26%, 3.36% of them are misclassified as “HVLA”, “LVHA”, and “LVLA” states, respectively. Third, comparing with the other three methods, MSFBEL shows improvement on each of the four emotional state. For instance, as shown in Figure 8, MSFBEL exceeds the accuracies of SVM, KNN, and SSFBEL on the “neutral” state recognition by 7.25%, 8.42%, and 5.37%, respectively. Further, we notice that “neutral” state always gets the highest accuracies on the four methods, which can deduce that it is the easiest emotional state to be identified. As displayed in Figure 9, MSFBEL exceeds the accuracies of other three methods on the “HVHA” state recognition by 14.44%, 10.19%, and 1.15%, respectively.

## 4. Conclusions and Future Work

In this paper, a new frequency bands ensemble method (MSFBEL) was proposed to recognize the emotional states from EEG data. The main advantages of MSFBEL are that (1) It re-organizes all frequency bands into different scales (several local scales and one global scale) to extract features and classify. (2) It combines the results of different scales by learning an adaptive weight to further improve emotion classification performance. Extensive experiments were conducted on the “SEED IV” and “DEAP” data set to evaluate the performance of MSFBEL. The results demonstrate that the scale of frequency bands influences the emotion recognition rate, while the global scale that directly concatenating all frequency bands cannot always guarantee to obtain the best emotion recognition performance. Moreover, the results also illustrate that different scales of frequency bands provide complementary information to each other, and the proposed adaptive weight learning method can effectively fuse these information. The results indicate the effectiveness of our proposed MSFBEL model in emotion recognition task.

In the future, we will focus on the following problem not covered in this paper. We will explore the cross subject or cross session domain adaptation problem, so that we may try to find the optimal scale of frequency bands for EEG-based emotion recognition.

## Figures and Tables

**Table 1:** The accuracies (%) of different scales of frequency bands on session 1 of “SEED IV” by using SSFBEL.

Subject	Local Scale				Global Scale
	Scale = 1	Scale = 2	Scale = 3	Scale = 4	Scale = 5
1	**84.85**	73.43	80.19	76.69	83.68
2	96.04	**100**	**100**	90.68	**100**
3	**82.75**	72.26	81.82	67.60	82.05
4	**100**	92.07	89.74	89.74	89.74
5	68.30	73.66	69.00	75.29	**79.72**
6	65.73	**79.25**	78.55	72.73	66.67
7	**97.20**	96.04	69.93	76.92	79.72
8	65.03	79.49	**83.22**	70.63	77.86
9	**93.47**	**93.47**	**93.47**	**93.47**	**93.47**
10	**83.45**	74.13	63.64	65.27	66.20
11	80.19	69.00	**81.82**	75.29	76.46
12	**79.95**	77.62	77.62	74.13	73.66
13	66.67	75.76	**80.65**	76.92	76.69
14	72.49	**84.15**	77.39	70.86	70.86
15	**88.58**	82.28	81.59	85.55	83.92
Average	**81.65**	81.51	80.57	77.45	80.05

**Table 2:** The accuracies (%) of different scales of frequency bands on session 2 of “SEED IV” by using SSFBEL.

Subject	Local Scale				Global Scale
	Scale = 1	Scale = 2	Scale = 3	Scale = 4	Scale = 5
1	64.06	67.97	**76.28**	72.13	**76.28**
2	**97.56**	91.69	**97.56**	96.33	88.51
3	**91.20**	85.82	88.75	**91.20**	**91.20**
4	82.89	**88.75**	84.11	**88.75**	**88.75**
5	80.93	82.40	**88.51**	**88.51**	**88.51**
6	88.75	92.18	**94.13**	85.09	89.49
7	97.31	**97.56**	92.91	**97.56**	**97.56**
8	71.15	78.24	**87.04**	78.24	80.93
9	**88.75**	75.79	73.11	73.35	73.11
10	88.75	**94.13**	92.91	**94.13**	**94.13**
11	69.68	**82.64**	80.68	78.00	80.68
12	77.26	79.95	**85.57**	71.39	79.95
13	78.97	73.11	72.37	**79.22**	75.79
14	74.82	73.11	80.68	**86.55**	82.40
15	**100**	94.13	91.69	91.69	94.13
Average	83.47	83.83	**85.75**	84.81	85.43

**Table 3:** The accuracies (%) of different scales of frequency bands on session 3 of “SEED IV” by using SSFBEL.

Subject	Local Scale				Global Scale
	Scale = 1	Scale = 2	Scale = 3	Scale = 4	Scale = 5
1	60.32	78.82	80.16	**80.43**	69.97
2	**89.01**	**89.01**	**89.01**	**89.01**	**89.01**
3	56.57	60.59	61.13	56.57	**78.55**
4	89.01	80.16	89.01	**91.96**	89.01
5	64.34	72.12	72.12	**85.79**	72.12
6	70.24	80.97	88.47	**89.01**	**89.01**
7	89.01	**94.91**	84.45	70.24	84.99
8	63.00	71.58	78.55	75.87	**86.33**
9	54.96	62.73	62.73	62.73	**66.49**
10	64.61	64.61	69.17	64.61	**74.80**
11	**67.29**	56.57	61.13	61.13	61.13
12	**54.96**	49.06	49.06	53.62	53.62
13	61.13	**64.34**	61.13	61.13	61.13
14	79.36	70.24	**95.44**	89.01	**95.44**
15	78.55	78.55	78.55	78.55	**78.82**
Average	69.49	71.62	74.67	73.98	**76.79**

**Table 4:** The accuracies of different scales of frequency bands on the “DEAP” by using SSFBEL (%).

Subject	Local Scale			Global Scale
	Scale = 1	Scale = 2	Scale = 3	Scale = 4
1	60.21	77.92	80.42	**86.88**
2	41.04	50.42	51.67	**57.92**
3	53.13	60.63	62.50	**73.75**
4	55.42	60.21	**67.29**	63.54
5	48.54	58.33	58.54	**68.33**
6	58.54	67.50	72.71	**77.71**
7	45.63	60.00	60.83	**71.88**
8	45.63	65.63	63.54	**75.00**
9	47.92	60.83	63.75	**67.08**
10	65.42	**78.04**	72.92	75.92
11	42.92	56.88	58.33	**64.38**
12	51.25	**64.79**	62.71	64.21
13	73.13	74.17	**80.17**	77.58
14	55.00	63.96	**76.04**	72.50
15	71.04	79.79	80.42	**84.58**
16	67.08	82.29	**88.75**	87.71
17	48.33	65.63	66.67	**77.08**
18	49.58	69.58	70.63	**81.67**
19	47.92	57.08	57.50	**64.17**
20	58.33	69.79	73.54	**77.50**
21	48.54	60.21	61.25	**67.71**
22	42.71	**54.04**	50.21	51.75
23	64.38	78.75	83.75	**87.29**
24	54.17	59.79	**63.54**	61.67
25	58.96	66.67	68.75	**70.83**
26	51.88	64.79	66.25	**70.21**
27	62.50	63.54	64.17	**67.50**
28	49.79	73.33	72.92	**80.21**
29	47.29	62.29	64.17	**72.71**
30	44.17	62.50	64.38	**72.50**
31	46.67	62.92	66.88	**73.75**
32	49.38	56.67	**68.54**	65.00
Average	53.33	65.28	67.62	**72.20**

**Table 5:** The comparison of different methods on the “SEED IV” (%).

Subject	Session 1				Session 2				Session 3			
	SVM	KNN	SSFBEL	MSFBEL	SVM	KNN	SSFBEL	MSFBEL	SVM	KNN	SSFBEL	MSFBEL
1	73.43	65.97	**84.85**	80.19	**76.28**	75.06	**76.28**	**76.28**	63.27	57.91	69.97	**80.16**
2	86.01	**100**	96.04	**100**	**97.56**	94.62	**97.56**	**97.56**	81.77	70.24	**89.01**	**89.01**
3	75.99	75.76	**82.75**	73.66	75.06	81.91	88.75	**91.20**	56.57	49.06	**78.55**	**78.55**
4	83.22	79.25	**100**	**100**	71.15	84.11	84.11	**88.75**	**94.91**	89.01	89.01	91.42
5	52.68	59.21	68.30	**75.29**	82.64	77.26	**88.51**	85.57	**78.02**	76.14	72.12	75.87
6	59.44	54.55	65.73	**66.67**	64.55	80.44	**94.13**	93.40	69.71	**89.01**	**89.01**	**89.01**
7	52.45	71.33	**97.20**	**97.20**	91.69	97.56	92.91	**99.27**	85.25	80.16	84.99	**94.91**
8	77.16	77.16	65.03	**79.49**	66.99	**88.02**	87.04	87.04	**89.54**	81.23	86.33	86.33
9	83.22	87.41	93.47	**100**	73.11	67.24	73.11	**74.82**	62.73	62.73	**66.49**	**66.49**
10	45.22	58.28	**83.45**	73.66	85.33	91.20	**92.91**	91.69	68.36	**75.07**	74.80	64.88
11	70.16	72.49	**80.19**	79.72	**80.93**	66.99	80.68	80.68	61.13	49.06	61.13	**67.29**
12	75.76	71.33	**79.95**	77.62	57.21	61.37	**85.57**	83.86	49.06	**59.52**	53.62	54.96
13	**83.68**	60.14	66.67	77.62	78.48	62.59	72.37	**87.29**	**61.13**	**61.13**	**61.13**	**61.13**
14	64.34	66.90	**72.49**	**72.49**	77.75	74.33	80.68	**86.55**	86.60	54.96	**95.44**	**95.44**
15	85.55	64.80	**88.58**	87.65	85.33	**100**	91.69	94.13	68.36	**78.82**	**78.82**	78.55
Average	71.22	70.97	81.65	**82.75**	77.60	80.18	85.75	**87.87**	71.76	68.94	76.69	**78.27**
Std	12.94	11.58	11.57	**10.92**	10.07	12.05	7.52	**6.83**	13.03	13.08	**12.02**	12.45

**Table 6:** The comparison of different methods on the “SEED IV” (%).

Subject	SVM	KNN	SSFBEL	MSFBEL	Subject	SVM	KNN	SSFBEL	MSFBEL
1	59.79	75.83	86.88	**87.71**	17	56.04	59.79	**77.08**	75.63
2	43.75	57.50	57.92	**59.79**	18	55.42	72.29	81.67	**82.92**
3	52.71	66.04	73.75	**75.63**	19	61.25	64.58	64.17	**65.63**
4	54.17	62.50	63.54	**70.21**	20	61.04	62.92	77.50	**79.38**
5	42.71	55.63	**68.33**	**68.33**	21	54.38	62.50	67.71	**69.58**
6	49.58	61.88	77.71	**80.83**	22	36.88	50.21	51.75	**63.96**
7	63.13	71.88	71.88	**73.54**	23	62.92	76.25	**87.29**	85.00
8	51.25	60.21	75.00	**76.04**	24	56.67	60.42	61.67	**64.58**
9	52.50	56.88	67.08	**67.29**	25	52.29	63.75	70.83	**74.38**
10	55.83	60.21	75.92	**77.92**	26	52.50	55.42	**70.21**	69.79
11	34.38	51.67	64.38	**70.21**	27	62.08	**68.75**	67.50	**68.75**
12	56.46	61.25	64.21	**66.04**	28	48.33	61.04	80.21	**82.92**
13	68.13	69.38	77.58	**81.88**	29	53.33	60.42	72.71	**73.54**
14	54.79	59.38	72.50	**73.54**	30	46.46	61.67	72.50	**73.96**
15	59.79	70.42	**84.58**	84.17	31	52.71	64.17	**73.75**	**73.75**
16	57.08	68.33	87.71	**88.96**	32	46.88	54.17	65.00	**69.38**
**Method**		**SVM**		**KNN**		**SSFBEL**		**MSFBEL**	
**Average**		53.60		62.73		72.20		**74.23**	
**Std**		7.32		**6.34**		8.42		7.23	

## Appendix A

Below we give the solution of the problem (11). We alternately optimize w and v, which updates one variable with the other fixed.

(1) Update v with w fixed. By taking the derivative of objective (11) with respect to v and setting it to zero, we get

(A1) $$v=w+\frac{b+\beta -Mw}{\mu }.$$

(2) Update w with v fixed. When v is fixed, objective (11) becomes

(A2) $$\min_{w\geq 0,w^{T}1_{s}=1}v^{T}Mw+\frac{\mu }{2}{∥w-v+\frac{\beta }{\mu }∥}_{2}^{2}.$$

By completing the square form with respect to w, we rewrite the above objective as

(A3) $$\min_{w\geq 0,w^{T}1_{s}=1}\frac{\mu }{2}{∥w-v+\frac{\beta +M^{T}v}{\mu }∥}_{2}^{2}.$$

Problem (A3) is a typical Euclidean projection on the simplex [33,41], and we solve it as below. By denoting $g=v-\frac{\beta +M^{T}v}{\mu }$, the Lagrangian function of problem (A3) is

(A4) $$L\left(w,\gamma ,\eta \right)=\frac{1}{2}{∥w-g∥}_{2}^{2}-\gamma \left(w^{T}1_{s}-1\right)-\eta^{T}w,$$

where γ and $\eta \in R^{s}$ are the Lagrangian multipliers. Suppose that $w^{*}$ is the optimal solution, $\gamma^{*}$ and $\eta^{*}$ are the corresponding Lagrangian multipliers. According to KKT condition [42], we get the equations for every *j*(j=1,2,⋯,s) as follows

(A5) $$\left\{\begin{array}{c} w_{j}^{*}-g_{j}-\gamma^{*}-\eta_{j}^{*}=0\\ w_{j}^{*}\geq 0\\ \eta_{j}^{*}\geq 0\\ w_{j}^{*}\eta_{j}^{*}=0. \end{array}\right.$$

The first equation of Equation (A5) can be reformulated into the following form

(A6) $$w^{*}-g-\gamma^{*}1_{s}-\eta^{*}=0.$$

With the constraint $w^{T}1_{s}=1$, the above equation becomes

(A7) $$\gamma^{*}=\frac{1-1_{s}^{T}g-1_{s}^{T}\eta^{*}}{s}.$$

By substituting Equation (A7) into Equation (A6), we obtain

(A8) $$w^{*}=g-\frac{1_{s}^{T}g}{s}1_{s}+\frac{1}{s}1_{s}-\frac{1_{s}^{T}\eta^{*}}{s}1_{s}+\eta^{*}.$$

Suppose ${\bar{\eta }}^{*}=\frac{1_{s}^{T}\eta^{*}}{s}$ and $h=g-\frac{1_{s}^{T}g}{s}1_{s}+\frac{1}{s}1_{s}$, Equation (A8) becomes

(A9) $$w^{*}=h+\eta^{*}-{\bar{\eta }}^{*}1_{s}.$$

Hence, for every *j*, we obtain

(A10) $$w_{j}^{*}=h_{j}+\eta_{j}^{*}-{\bar{\eta }}^{*}.$$

According to Equations (A5) and (A10), we obtain $h_{j}+\eta_{j}^{*}-{\bar{\eta }}^{*}={\left(h_{j}-{\bar{\eta }}^{*}\right)}_{+}$, where ${\left(f\left(\cdot \right)\right)}_{+}=max\left(f\left(\cdot \right),0\right)$. Hence, we have

(A11) $$w_{j}^{*}={\left(h_{j}-{\bar{\eta }}^{*}\right)}_{+}.$$

Once the optimal ${\bar{\eta }}^{*}$ is determined, the optimal solution $w^{*}$ will be obtained from Equation (A11). Similarly, Equation (A10) can also be reformulated into $\eta_{j}^{*}=w_{j}^{*}+{\bar{\eta }}^{*}-h_{j}$ such that $\eta_{j}^{*}={\left({\bar{\eta }}^{*}-h_{j}\right)}_{+}$. Thus, ${\bar{\eta }}^{*}$ can be computed by

(A12) $${\bar{\eta }}^{*}=\frac{1}{s}\sum_{t=1}^{s}{\left({\bar{\eta }}^{*}-h_{j}\right)}_{+}.$$

Defining a function as

(A13) $$f\left(\bar{\eta }\right)=\frac{1}{s}\sum_{t=1}^{s}{\left(\bar{\eta }-h_{j}\right)}_{+}-\bar{\eta }.$$

When Equation (A13) equals to zero, the optimal ${\bar{\eta }}^{*}$ can be obtained by Newton method [43] to find the root of Equation (A13) as following

(A14) $${\bar{\eta }}_{p+1}={\bar{\eta }}_{p}-\frac{f({\bar{\eta }}_{p})}{f^{\prime }\left({\bar{\eta }}_{p}\right)}.$$
